# Triplex Hybridization-Based Nanosystem for the Rapid Screening of Pneumocystis Pneumonia in Clinical Samples

**DOI:** 10.3390/jof6040292

**Published:** 2020-11-17

**Authors:** Luis Pla, Anna Aviñó, Ramón Eritja, Alba Ruiz-Gaitán, Javier Pemán, Vicente Friaza, Enrique J. Calderón, Elena Aznar, Ramón Martínez-Máñez, Sara Santiago-Felipe

**Affiliations:** 1 Instituto Interuniversitario de Investigación de Reconocimiento Molecular y Desarrollo Tecnológico, Universitat Politècnica de València, Universitat de València, Camino de Vera s/n, 46022 Valencia, Spain; plablas@upv.es (L.P.); sasanfe@upvnet.upv.es (S.S.-F.); 2 Unidad Mixta de Investigación en Nanomedicina y Sensores, Instituto de Investigación Sanitaria La Fe, Universitat Politècnica de València, 46022 Valencia, Spain; 3 CIBER de Bioingeniería, Biomateriales y Nanomedicina (CIBER-BBN), Spain; aaagma@cid.csic.es (A.A.); recgma@cid.csic.es (R.E.); 4 Institute for Advanced Chemistry of Catalonia (IQAC), CSIC, Jordi Girona 18-26, 08034 Barcelona, Spain; 5 Grupo Acreditado de Infección Grave, Instituto de Investigación Sanitaria La Fe and Servicio de Microbiología, Hospital Universitari i Politècnic La Fe, Avenida Fernando Abril Martorell, 46026 Valencia, Spain; alba_ruiz@iislafe.es (A.R.-G.); peman_jav@gva.es (J.P.); 6 Instituto de Biomedicina de Sevilla, Hospital Universitario Virgen del Rocío/Consejo Superior de Investigaciones Científicas/Universidad de Sevilla, 41013 Sevilla, Spain; vfriaza-ibis@us.es (V.F.); sandube@cica.es (E.J.C.); 7 Centro de Investigación Biomédica en Red de Epidemiología y Salud Pública (CIBERESP), Spain; 8 Unidad Mixta UPV-CIPF de Investigación en Mecanismos de Enfermedades y Nanomedicina, Universitat Politècnica de València, Centro de Investigación Príncipe Felipe, 46012 Valencia, Spain

**Keywords:** nanoporous anodic alumina, *Pneumocystis jirovecii*, molecular gates, oligonucleotides, biosensor

## Abstract

Pneumocystis pneumonia (PcP) is a disease produced by the opportunistic infection of the fungus *Pneumocystis jirovecii*. As delayed or unsuitable treatments increase the risk of mortality, the development of rapid and accurate diagnostic tools for PcP are of great importance. Unfortunately, current standard methods present severe limitations and are far from adequate. In this work, a time-competitive, sensitive and selective biosensor based on DNA-gated nanomaterials for the identification of *P. jirovecii* is presented. The biosensor consists of a nanoporous anodic alumina (NAA) scaffold which pores are filled with a dye reporter and capped with specific DNA oligonucleotides. In the presence of *P. jirovecii* genomic DNA, the gated biosensor is open, and the cargo is delivered to the solution where it is monitored through fluorescence spectroscopy. The use of capping oligonucleotides able to form duplex or triplex with *P. jirovecii* DNA is studied. The final diagnostic tool shows a limit of detection (LOD) of 1 nM of target complementary DNA and does not require previous amplification steps. The method was applied to identify DNA from *P. jirovecii* in unmodified bronchoalveolar lavage, nasopharyngeal aspirates, and sputum samples in 60 min. This is a promising alternative method for the routinely diagnosis of Pneumocystis pneumonia.

## 1. Introduction

*Pneumocystis jirovecii* is an opportunistic fungus that can cause asymptomatic or mild infection in healthy people and fulminating pneumonia in immunocompromised individuals [1]. *Pneumocystis* spp. can affect many species of mammals (rodent, horses, and primates) and presents strong specificity for host species. The fungus was identified as a human pathogen in the 1940s when it was detected in the lungs of children affected with interstitial pneumonia of plasmatic cells [2]. Usually, in humans, the pathogen is disseminated from person to person producing asymptomatic or subclinical infection; however, when it is transmitted to an immunocompromised host, a severe pneumonia can take place (Pneumocystis pneumonia, PcP) [3]. In the 1980s, the high incidence of acquired immunodeficiency syndrome (AIDS) lead to a markedly interest in PcP due to the high incidence in those patients. Nowadays, the widespread use of immunosuppressive treatments such as corticosteroid therapies, chemotherapies, or biological therapies, has increased the incidence of *P. jirovecii* infections. In addition, recent studies have provided evidence of transplacental transmission in humans and stablished a relation between *P. jirovecii* colonization in preterm infants and neonatal Respiratory Distress Syndrome risk [4,5].

In the last decade, several published works have allowed a high progress in the knowledge of the *Pneumocystis* pathobiology; however, morbidity and mortality due to PcP still remains high, especially in developing countries. One of the main challenges in the diagnosis of PcP is the absence of a specific clinical manifestation, which difficult diagnosis and makes it stand on non-specific symptoms [6]. Therefore, a proper diagnosis of *Pneumocystis* pneumonia involves correct recognition of the fungus. Unfortunately, the current standard methods for *P. jirovecii* detection are far from adequate, and further aggravated by the impossibility to keep continuous fungus ex vivo axenic culture [7].

Classical methodologies for the identification of *P. jirovecii* comprise microscopic observation of stained biological samples that reveal the characteristic morphology of *Pneumocystis* cystic and/or trophic forms. These techniques provide sensitivity and specificity values between 60–75% and 80–90%, respectively; however, they remain time-consuming and require high experienced staff and specific equipment [8,9,10]. Some alternatives for PcP diagnosis involve the detection of blood biomarkers that indicate the host-pathogen interaction, including (bio)molecules such as (1,3)-β-d-glucan (BG), lactate dehydrogenase (LDH), Krebs von den Lungen-6 antigen (KL-6), and adenosylmethionine (SAM) [10]. Nonetheless, despite their good sensitivity (70–95%), the detected metabolites are not strictly specific to *P. jirovecii* infection, which significantly reduces the specificity values (50–70%) [10]. On the other hand, serological methods detect the antibodies produced by the patient as consequence of the presence of the fungus, but despite their greater selectivity, they are expensive, require long-time procedures, and there is still a need to better characterize the immune response to obtain better standardized protocols [9,11,12].

Among molecular techniques, PCR assays have revealed to be highly efficient, allowing even the early detection of the fungus in samples from patients that were confirmed negative by microscopic examination [8]. During the last 15 years, numerous PCR techniques for the detection of genomic DNA from *P. jirovecii* have been reported, obtaining sensitivity and specificity values close to 90–100%. The most remarkable studies employ both conventional PCR and its variants nested-PCR, and real time PCR, and they are applied to recognize target gene such as the mitochondrial large subunit RNA (mtLSUrRNA), the internally transcribed spacer (ITS) or 18S or 5S ribosomal RNA (18S rRNA or 5S rRNA) [8]. However, despite its great potential, PCR is not exempt from limitations such us high susceptibility to polymerase inhibitors or cross contamination [13].

In the last decades, new diagnostic platforms based upon nanotechnology have become a reality thanks to their ability to provide sensitive, accurate and rapid results. For instance, nanotechnology has burst into *Pneumocystis* diagnosis by developing serological biosensors that uses gold nanoparticles (AuNPs) to detect anti-*P. jirovecii* antibodies by a colorimetric assay in a lateral flow immunoassay [14,15]. Nevertheless, the development of nanosensors for PcP detection is still an incipient area. However, this is a stimulating field of research and recent advances have demonstrated that the design of new hybrid organic–inorganic systems in a single entity can benefit from both, the potential of nanomaterials and the recognition, selectivity, and sensitivity properties of biomolecules. In that regard, several hybrid systems have been extensively applied in sensing and drug delivery applications [16,17,18,19,20,21,22,23].

Among the great variety of nanomaterials available, nanoporous anodic alumina (NAA) has been widely used because of its biocompatibility, high surface area, high loading capacity, and easy modification of the surface. Moreover, NAA supports can be easily prepared by cost-competitive and well-known production techniques [24]. NAA have additionally been used to develop capped stimuli-responsive systems, in which the mesoporous support is loaded with a cargo and capped with a biomolecule, so that only target molecules are able to induce cargo release. In NAA, oligonucleotides as capping systems have recently been demonstrated to be excellent candidates to detect and quantify a range of molecules and biomolecules [24,25]. Some of these tools rely on the use of DNA as caps and base their recognition mechanism on duplex hybridization interactions (DNA-DNA or DNA-RNA) between the probe and the target. Nevertheless, innovative triple-helix hybridization formats based on Hoogsteen and reverse-Hoogsteen base pairs to the Watson-Crick duplex are also interesting approaches that have demonstrated increased recognition efficiency.

Triplex formation is observed at certain polypurine-polypyrimidine sequences that are widely found in the human genome, particularly at promoter regions [26,27,28,29]. Several published studies have described the triplex-stabilizing features of 8-aminopurines and the use of parallel and antiparallel tail-clamps to improve the efficacy of triplex recognition with complementary RNA and DNA targets [30,31]. On that regard, this triplex hybridization has been effectively applied to develop biosensors. For example, Carrascosa et al. and Aviñó et al. developed a sensitive and label-free method for the detection of an mRNA from Listeria and miRNA-145 by the formation of triplex hybridization structures in SPR biosensors [32]. More recently, Wei et al. have reported a method that combines the formation of triplex DNA structures with further amplification reactions for the sensitive detection of microRNAs [33] and some of us have developed an oligonucleotide-gated mesoporous support for the identification of miRNA-145 based on the formation of triple-helix structure, allowing a sensitivity as low as 2.5 pM and accurate qualitative determination in human serum samples [34].

Based on the above, we report herein two probes for the specific identification of *P. jirovecii* using gated nanomaterials. The diagnostic tool is based in NAA, whose pores are loaded with the fluorophore rhodamine B and blocked with different oligonucleotide probes (single or hairpin strands, see Scheme S1) able to form duplex or triplex structures with the target gene of mtLSUrRNA of *P. jirovecii* DNA. Best analytical performances are obtained for the triplex hybridization with a LOD of 1 nM in less than one hour without previous DNA amplification. Likewise, this method detects *P. jirovecii* DNA in unmodified sputum, nasopharyngeal aspirates (NPA), and bronchoalveolar lavage (BAL) samples, showing its great potential in point-of-care applications.

## 2. Materials and Methods

### 2.1. General Techniques

A ZEISS Ultra 55 microscope was employed to perform field emission scanning electron microscopy (FSEM) and energy dispersive X-ray spectroscopy (EDX) analyses. Fluorescence spectroscopy measurements were carried out on a Synergy H1 microplate reader (BioTek, Winooski, VT, USA).

### 2.2. Chemicals

Tris(hydroxymethyl)aminomethane (TRIS), hydrochloric acid, (3-aminopropyl)triethoxysilane (APTES), and rhodamine B, were obtained from Sigma-Aldrich Quimica (Madrid, Spain). NAA scaffolds were purchased from InRedox (Longmont, CO, USA).

### 2.3. Synthesis of Oligonucleotides

In this study it was used the gene encoding the mitochondrial large-subunit of *P. jirovecii* (mt LSU rRNA). Design of oligonucleotides and in silico analysis is described in the Electronic Supplementary Information (Scheme S1). Table 1 shows the sequences of the oligonucleotides employed in this study. The synthesis took place by the well-defined phosphoramidite solid phase protocol [32]. For this, oligonucleotides were assembled on controlled pore glass (CPG) scaffolds by consecutive incorporation of the suitable phosphoramidites employing an automated Applied Biosystems 394 DNA synthesizer (Foster City, CA, USA). Then, the scaffolds were treated overnight with concentrated ammonia at 55 °C. Finally, synthetized oligonucleotides were purified by Glen-PackTM DNA cartridges (Glen Research, Sterling, VA, USA) and mass spectrometry was used to analyze them (Table S1, ESI).

### 2.4. Synthesis of Nanomaterials S0, S1 (Duplex), S2 (Clamp), and S3 (Control)

For the synthesis of **S0**, 10 individual NAA scaffolds (2 mm of diameter each one) were submerged in a solution of rhodamine B in CH_3_CN (1 mM, 8 mL). The mixture was stirred at room temperature for 24 h. Then the functionalization of the surface with aminopropyl moieties was carried out by adding 40 µL per individual NAA scaffold of (3-aminopropyl)triethoxysilane (1.25 µM) and stirring the mixture at room temperature for 6 h. For the synthesis of the sensing probes **S1**, **S2,** and **S3**, different pieces of solid **S0** were capped by adding to each support 10 µL of the corresponding oligonucleotide (**O1, O2,** or **O3**, respectively) (100 µM) in a final volume of 250 µL of hybridization buffer (20 mM Tris-HCl, 37.5 mM MgCl_2_, pH 7.5). The mixtures were shaken for 60 min at 37 °C. The capped materials were rinsed with hybridization buffer to remove the unbounded oligonucleotide.

### 2.5. Release Kinetics

The response of the gated materials was determined by measuring the fluorescence of the dye released from the pores to the solution in the presence of the target complementary DNA. In a common experiment, two independent supports of each material **S1**, **S2,** and **S3** were immersed in 900 µL of hybridization buffer. Then, 100 µL of the complementary DNA (10 µM) was added to one of the supports while 100 µL of hybridization buffer was added to the other. Both solutions were stirred at 37 °C and aliquots were taken at scheduled times. Finally, dye delivery was determined by registering the fluorescence of the rhodamine B in the solution at 575 nm (λ_exc_ = 555 nm).

### 2.6. Real Media Experiments

The response of **S2** was assessed in a more realistic context. For that, 500 µL of sputum, bronchoalveolar lavage (BAL) and nasopharyngeal aspirate (NPA) samples were artificially inoculated with 100 µL of DNA from *P. jirovecii* (10 µM) and added to two independent **S2** supports in a final volume of 1 mL in hybridization buffer. Solutions were maintained at 37 ˚C and rhodamine B released from the pores was measured at 575 nm (λ_exc_ = 555 nm) after 60 min.

### 2.7. Response to Different Target Concentrations

The response of the material **S2** to different concentrations of the target DNA was assessed. For this, six independent supports were submerged separately in 900 µL of hybridization buffer and 100 µL of 10-fold diluted target solutions were added to each one, reaching final concentrations from 10^−5^ to 1 µM. After 60 min at 37 °C, the released rhodamine B from the porous was measured at 575 nm (λ_exc_ 555 nm).

### 2.8. Selectivity to Possible Interferents

The selectivity of the method was determined for **S2** by carrying out cargo delivery experiments in the presence of 100 µL of DNA (10 ng/µL) from other microorganisms (*Aspergillus* spp., *Candida albicans, Candida tropicalis, Candida auris, Schizosaccharomyces pompe,* and *Taphrina deformans*) in a final reaction volume of 1 mL of hybridization buffer. In the same experiment, 100 µL of complementary DNA from *P. jirovecii* at 10 ng/µL was used as a positive control and 100 µL of hybridization buffer as a negative control. Mixtures were maintained in agitation for 60 min at 37 °C and the delivered rhodamine B was determined by measuring the fluorescence in the solution (λ_exc_ = 555 nm, λ_em_ = 585 nm).

### 2.9. Validation of the Method in Clinical Samples

In order to determine the clinical applicability of the gated nanosensor, the performance of **S2** was evaluated in a more realistic media. First, the system was used to analyze 8 sputa and 4 bronchoalveolar lavages (BAL) from infected and non-infected patients from the Hospital Universitari i Politècnic La Fe. In another experiment, 21 nasopharyngeal aspirates (NPA) from colonized and non-colonized newborn infants from the Hospital Universitario Virgen del Rocío were analyzed using **S2** nanomaterials. For both assays, 250 µL of each sample were added to individual **S2** solids in a final volume of 400 µL of hybridization buffer. After 60 min at 37 °C, dye released from the pores to the solution was measured by fluorescence spectroscopy at 575 nm (λ_exc_ 555 nm).

## 3. Results and Discussion

### 3.1. Synthesis and Characterization of the Biosensors

**Figure 1:**
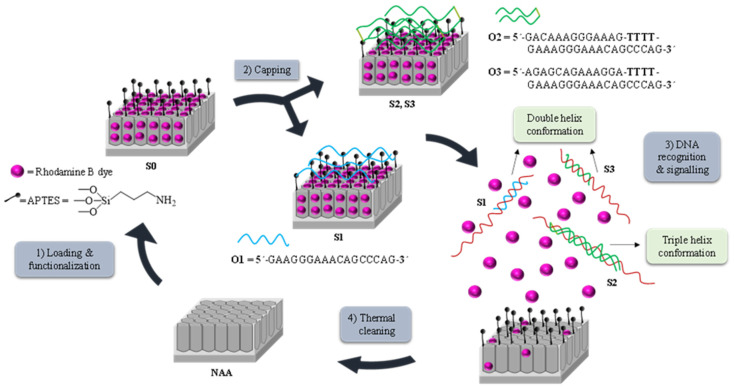
Representation of the preparation and sensing process: (1) Loading and functionalization of the nanoporous anodic alumina (NAA) support; (2) Capping with the oligonucleotides **O1**, **O2,** or **O3**, to obtain solids **S1**, **S2,** and **S3**, respectively; (3) Release of the cargo in the presence of *P. jirovecii* genomic DNA by forming (A) duplex structures when using solids **S1** or **S3** or (B) triplex hybridization structures when using **S2** and (4) Calcination prior to the reutilization of the scaffold.

Figure 1 depicts the synthetic process followed to obtain the biosensors and their performance in the presence of the target DNA. NAA scaffolds were selected as inorganic supports to develop the capped nanomaterials. First, pores were filled with the dye rhodamine B which has demonstrated excellent properties for the preparation of optical gated nanosensors applied to the detection of different analytes [18,19,20,21,22,23,24,25,26]. In a second step, the outer surface of the NAA support was functionalized with the linker (3-aminopropyl)triethoxysilane, yielding **S0**. At a neutral pH, aminopropyl moieties are partially protonated (and therefore positively charged) being able to electrostatically interact with negatively charged oligonucleotides. Based on this, the interaction of oligonucleotides **O1**, **O2,** or **O3** with the amino-functionalized **S0**, yielded the gated sensing solids **S1**, **S2,** and **S3**. **O1** consists of a single strand oligonucleotide that hybridizes with the DNA sequence forming a duplex structure, **O2** is a clamp that forms a triplex hybridization structure, whereas **O3** is also a clamp but results in a duplex conformation.

The recognition and detection mechanism of the loaded and capped **S1**, **S2,** and **S3** materials takes place as follows. In the absence of target analyte, the oligonucleotides **O1**, **O2,** or **O3**, electrostatically attached to the external surface of the inorganic scaffold, were expected to be bulky enough to block pores and to inhibit dye delivery. On the contrary, due to the higher affinity of the oligonucleotide probes for the target than for the aminopropyl moieties, it was expected that in the presence of the analyte, the capping oligonucleotides were selectively displaced from the surface resulting in DNA target-probe hybridization, pore opening and dye diffusion from the pores to the aqueous solution. Finally, the used solids were calcined to remove organic matter, allowing supports to be used again (Figure 1).

**Figure 2:**
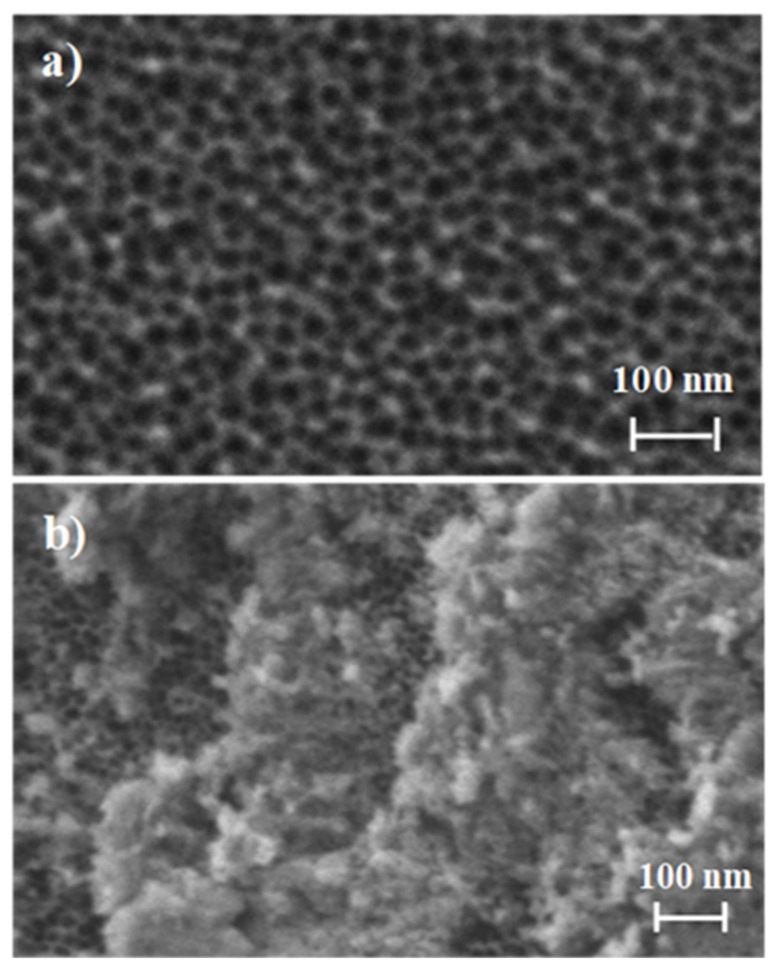
Representation of the preparation and sensing process: (**a**) Loading and functionalization of the NAA support; (**b**) Capping with the oligonucleotides.

The starting NAA supports were commercially obtained from InRedox^®^ (Longmont, CO, USA). The supports are anodic aluminum oxide (AAO) films grown on a 0.1 mm thick aluminum layer with a pore density of 9·10^11^ cm^−2^. Pore entrance has a funnel-like form which gradually decreases from a larger size (20–30 nm) at the top of the funnel to a 5 nm size at the end. Pores have a profundity of ca. 10 µm. The starting NAA scaffold and **S1**, **S2,** and **S3** were characterized following standard techniques such as FESEM and EDX analysis. FESEM images of the starting NAA scaffolds confirmed the expected non-ordered porous structure, whereas typical imagens of **S1**, **S2,** and **S3** showed the presence of an organic layer attributed to the capping oligonucleotides (Figure 2). Furthermore, in **S1**, **S2,** and **S3**, the porous structure of the NAA scaffolds can still be seen in some places where the surface was not completely covered, demonstrating that the loading, functionalization, and capping steps did not modify the NAA structure. This behavior agrees with that observed in previous studies carried out with the same or similar materials [35,36,37].

The organic content of the different prepared materials (**S0**, **S1**, **S2,** and **S3**) was studied by energy dispersive X-ray spectroscopy (EDX) (Table 2). After dye loading and surface functionalization with aminopropyl moieties, solid **S0** shows a high C/Al content, which was reduced in **S1**, **S2,** and **S3** due to the partial rhodamine B release that takes place during the gating process. Moreover, the existence of nitrogen in all final solids is indicative of the presence of aminopropyl groups and oligonucleotides. The occurrence of the latter is also confirmed by the presence of phosphorous atoms.

### 3.2. Delivery Kinetics

**Figure 3:**
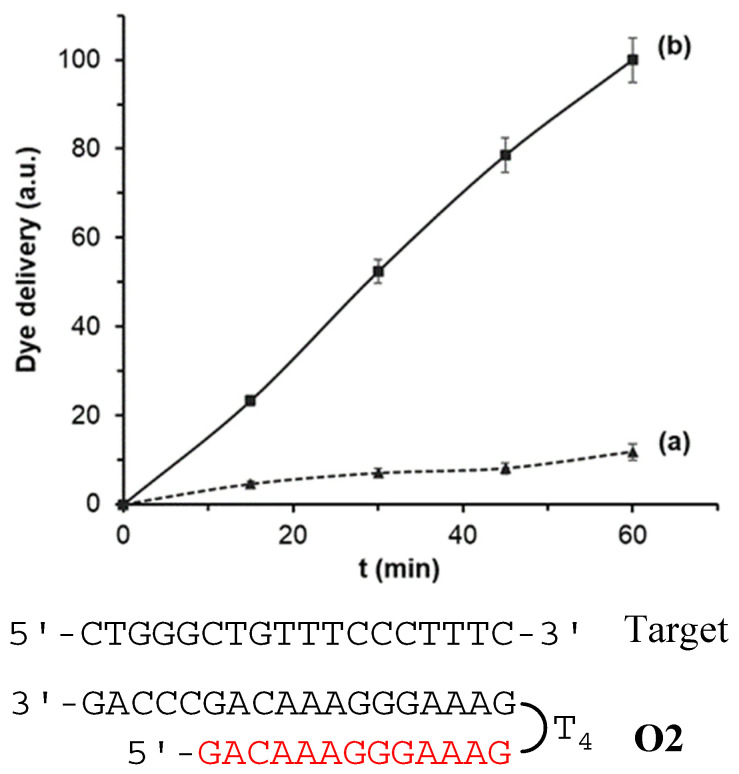
Rhodamine B delivery from **S2** (**a**) in the absence and (**b**) in the presence of 1 nM of complementary **O4** target sequence in a solution of hybridization buffer (20 mM Tris-HCl, 37.5 mM MgCl_2_, pH 7.5).

In a first step, the response of the nanoprobes **S1**, **S2,** and **S3** to the presence of the target sequence **O4** was studied. In a typical experiment, two individual solids of each material **S1**–**S3** were submerged in hybridization buffer. Then, 100 μL of the complementary oligonucleotide sequence (**O4**, 1 ng μL^−1^) were transferred to one of the supports while 100 µL of buffer were added to the other. Mixtures were stirred at 37 °C and at predetermined times (i.e., 15, 30, 45, and 60 min) the fluorescence of the solution was measured to quantify the amount of the dye delivered from the pores to the aqueous phase. As an example, Figure 3 shows the delivery profile for solid **S2**. When the target sequence **O4** was present, a remarkable dye delivery from the pores to the solution was observed as a result of the oligonucleotide-DNA hybridization to form a DNA triplex structure (curve b), pore opening and dye release. The maximum dye diffusion from the pores to the solution was reached at 60 min and this value was considered the 100% of delivered dye. On the contrary, in the lack of the **O4** sequence, a very low dye is released, what indicates tight pore blockage by the probes electrostatically attached to the scaffold surface (curve a).

Similar delivery profiles were observed for solids **S1** and **S3**, also indicating adequate performance for these solids in the stages of DNA recognition and dye release (Figure S1, ESI). In terms of blockage ability, all three developed systems were able to keep the pore entrances closed regardless the capping oligonucleotide was a clamp (**S2** and **S3**) or a single strand, obtaining in all cases a very low residual dye delivery (lower than 20%) in the absence of **O4** target sequence.

All three nanodevices were also able to deliver the cargo in the presence of target sequence **O4**. However, differences in terms of the amount of dye delivered were observed when comparing the formation of triplex (**S2**) and duplex hybridization (**S1** and **S3**) structures. The obtained results showed that nanomaterial **S2** provided a faster response and the highest cargo release, reaching a first order constant rate of the release kinetics of 1.70 and an amount of ca. 2.8 ng of delivered Rhodamine B, calculated by interpolating the fluorescence of the amount of dye diffused from the pores to the solution after 60 min into a calibration curve of Rhodamine B. On the other hand, for **S1** and **S3** materials, the uncapping mechanism and subsequent dye release was slower (constant rates of 1.16 and 1.14, respectively), and the amount of dye delivered at 60 min was lower (by ca. 20%). This in tentatively attributed to a more stable, favorable and rapid formation of the triplex structure than those obtained by the duplex conformations (**S1** and **S3**). These results agree with others found in the literature in which the efficiency and stability of triplex hybridization structures were higher than those obtained by duplex conformations [33,34] Therefore, further studies were carried out using **S2** as sensing material.

### 3.3. Analytical Performance: Sensitivity and Specificity Studies

**Figure 4:**
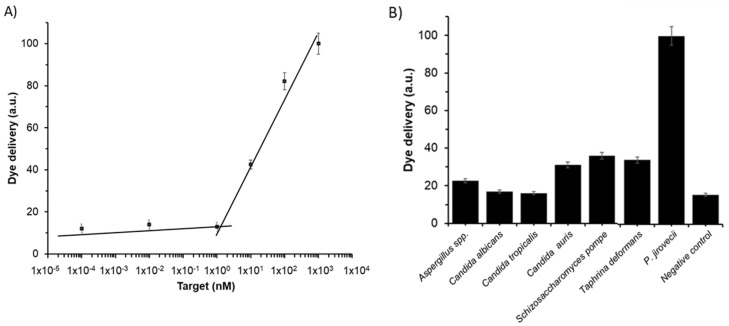
Release of rhodamine B from solid **S2** (**A**) in the presence of different concentrations of **O4** and (**B**) in the presence of 1 ng/µL of genomic DNA from *Aspergillus* spp., *C. albicans, C. tropicalis, C. auris, S. pompe, T. deformans, P. jirovecii*, and 100 µL of hybridization buffer as a negative control. Assays were performed at 60 min in hybridization buffer at pH 7.5.

New diagnostic tools should be evaluated in terms of limit of detection (LOD) and specificity. The LOD in **S2** was determined via the study of the response of the gated material to the presence of different amounts of target sequence **O4**. Based on the proposed sensing mechanism, the amount of released dye depends on the number of opened pores, which in turn depends on the number of target molecules that will induce the displacement of the capping molecules. Results depicted in Figure 4A show that the amount of delivered dye was directly proportional to the target DNA concentration, which agrees with the uncapping protocol detailed above. A LOD of 1 ± 0.1 nM was calculated based on the intersection point of the two slopes of the represented curve (Figure 4A). This LOD value is in the range of other reported protocols for the detection of *P. jirovecii* genomic DNA. Thus, for instance, several works based on the diagnosis of PcP by real time PCR reported LODs of 50–200 copies/µL, which corresponds to a DNA concentration range from ca. 250 pM to 1 nM. However, our system is faster, simpler, and requires no especial equipment or trained personnel [38,39,40,41,42,43].

The specificity of the system was evaluated by assessing the response of **S2** to the presence of genomic DNA from other fungi considered as possible interferents, such as *Aspergillus* spp., *C. albicans, C. tropicalis, C. auris, S. pompe*, and *T. deformans*. For that, the amount of dye delivered from the pores of seven independent **S2** supports was measured in the presence of 100 μL of DNA at 10 ng/µL of each possible interferent. Moreover, 100 µL of DNA from target **O4** at 10 ng/µL and 100 µL of hybridization buffer were added to **S2** as positive and negative control, respectively. As it is shown in Figure 4B, target sequence **O4**, present in *P. jirovecii* genomic DNA, is the only capable of induce a notable pore opening and rhodamine B release, whereas all the other fungi induced less response and lower dye release. That results confirmed the high selectivity of the system, even in the presence of DNA from *S. pompe* and *T. deformans*, which belongs to the same subphylum as Pneumocystis genus [44].

In a step forward, the robustness of the system was determined by evaluating the behavior of **S2** to the presence of the target sequence **O4** (0.1 ng/µL) in different respiratory fluids such as sputum, bronchoalveolar lavage (BAL) and nasopharyngeal aspirate (NPA) (Figure S2, ESI). In all media, the presence of **O4** produced a selective displacement of the oligonucleotide in **S2**, pore uncapping and dye delivery, whereas in the absence of target, a poor dye release was observed. A lower signal intensity was found in the sputum than in the BAL, NPA, and buffer media (60% vs. 100%), probably due to the higher complexity of the sputum samples. Nevertheless, the amount of dye delivered in all cases and the differences with respect to control samples (not having target **O4** sequence) is significant, allowing an accurate fungi DNA detection in all the samples (Figure S2, ESI). These results show that the proposed method may be a promising alternative to classical procedures for the detection of DNA from *P. jirovecii* in realistic environments.

In addition, it is worth mentioning that previous studies have demonstrated the possible re-use of these type of gated nanomaterials [25,36]. In this respect, used NAA supports were calcined to remove organic matter and reused up to 3 times for the detection of **O4** obtaining similar results.

### 3.4. P. jirovecii Detection in Clinical Samples

Once the method was analytically studied, a clinical validation is required to determine the real utility of the sensing material **S2** as a tool for the diagnosis of PcP produced by the infection of *P. jirovecii* as a possible alternative to existing conventional methods. The gold standard procedure used in most hospitals to diagnose PcP uses extraction of the genomic DNA from the fungus and its subsequent amplification by PCR techniques.

In the present study, 12 respiratory samples consisting of sputum and BAL samples from infected and non-infected patients from Hospital Universitari i Politècnic La Fe, were analyzed using **S2** material and the hospital reference method (i.e., PCR). For that, samples were isolated from patients with respiratory symptoms as part of routine diagnosis treatment. All samples were analyzed by PCR for the detection of *P. jirovecii* genomic DNA. Extraction of DNA from the samples was carried out by the QIAamp DNA Blood and Tissue kit and PCR reactions were performed using the VIASURE *P. jirovecii* Real Time PCR Detection Kit. Following this procedure, ten samples were confirmed as positive and two resulted negative. In parallel, the same samples (i.e., unmodified sputum or BAL samples) were tested using **S2**. For that, 12 individual **S2** supports were immersed in 150 μL of hybridization buffer, then 250 μL of each raw sample were directly added without any previous treatment and the fluorescence in the solution, due to rhodamine delivery, was measured after 60 min. Results were considered positives when the fluorescence intensity was higher than the average fluorescence of the negative controls plus three times their standard deviation. As shown in Table 2 (samples 1 to 12), the results show a total coincidence between both methods, obtaining a sensitivity and a specificity of the 100%, and therefore, positive and negative predictive values of 100%, demonstrating an excellent analytical and clinical results when using **S2** as diagnostic tool.

On the other hand, **S2** was employed to evaluate 21 NPA samples from newborn infants with and without primary *Pneumocystis* infection from Hospital Universitario Virgen del Rocío. First, all samples were analyzed by Nested-PCR of the *Pneumocystis* mtLSU rRNA gene as described elsewhere [6]. Briefly, DNA from *P. jirovecii* was extracted using a QIAmp DNA Mini Kit with proteinase K digestion at 56 °C. In the first round of amplification, the external primers pAZ102-E and pAZ102-H were used. This yielded a 346-base pair (bp) fragment. The second round utilized the internal primers pAZ102-X and pAZ102-Y and yielded a 260-bp product. Both rounds of PCR comprised 35 amplification cycles. Specific amplification was corroborated by melting curve analysis (Tm: 76–77 °C) and amplicons were analyzed by electrophoresis on a 1.5% agarose gel containing ethidium bromide. Following this procedure, 11 samples were confirmed as positive and 10 resulted negative.

Nested-PCR positive samples were quantified by qPCR. Real-time quantitative PCR was performed in a CFX96 real time system (BIO-RAD) using AceQ qPCR SYBR^®^ Green Master Mix (Vazyme) in a final volume of 10 µL containing 0.2 mM of primers pAZ102-X and pAZ102-Y and 2 µL of DNA sample. Serial dilutions of the target sequence cloned in the pGEM-T Easy Vector (Promega) were used to generate standard curves. After quantification, all the samples showed less than 10^2^ copies/µL, which is equivalent to less than 25,000 copies per mL of NPA. That concentration confirms the *P. jirovecii* colonization condition and agrees with normal concentrations found in NPA samples from newborns with primary *Pneumocystis* infection [45].

In parallel, the same samples (i.e., unmodified NPA samples) were analyzed using **S2**. For that, 21 individual **S2** supports were submerged in 150 μL of hybridization buffer and 250 μL of of each raw sample were directly added without any previous treatment. Finally, the fluorescence of the solution was measured after 60 min and results were considered positives when the fluorescence intensity was higher than the average fluorescence of the negative controls plus three times their standard deviation. As it is depicted in Table 3 (samples 13 to 33), results showed the coincidence between two methods in 16 samples and a total of 5 incongruent values that corresponded to two false positives and three false negatives, gave a sensitivity of 70%, a specificity of 78%, and positive and negative predictive values of 78% and 70%, respectively. The incongruences observed between the results obtained by PCR and **S2** materials might be due several causes such as sample handling, DNA degradation or the very low concentration of pathogen present in NPA samples (calculated as less than 25,000 copies per mL of NPA). Nevertheless, it is noteworthy the high competitiveness of the gated biosensor as rapid diagnostic tool compared to the standard reference procedures, additionally providing easiness to use and lower cost, and avoiding sample treatments such as DNA extraction steps or amplification reactions [46].

## 4. Conclusions

Early diagnosis of PcP is decisive for a suitable treatment and for a better prognosis. In that regard, rapid and accurate diagnostic tools are important for an early identification of the pathogen and to reduce the risk of mortality associated. However, standard diagnosis of PcP in remote or less developed countries or in resource-limited settings is difficult as usually it is necessary the use of costly well-equipped laboratories and trained personnel. In this scenario, the design low-cost easy-to-use diagnostic systems and point-of-care devices from readily accessible bio-fluids constitute a potential solution. In this study, a new method for the detection of *P. jirovecii* genomic DNA by using gated nanomaterials is proposed. In the developed tool, the pores of a NAA scaffold are loaded with a dye and gated with oligonucleotides that selectively recognizes a sequence of the genomic DNA from the fungi. The sensing mechanism relies in the recognition between the gating oligonucleotides and the *P. jirovecii* genomic DNA that induces a displacement of the capping oligonucleotide, pore uncapping and cargo delivery. In addition, the use of different gating oligonucleotides in the form of single strands or clamps to obtain duplex or triple-helix hybridization structures is also studied. From the three evaluated nanomaterials (**S1**, **S2,** and **S3**), the solid **S2** provides the highest rhodamine B release and the faster response, probably due to more efficient formation of a triplex with respect to duplex conformations (**S1** and **S3**). **S2** showed a LOD 1 nM in hybridization buffer, which is equivalent to other state-of-the-art reported detection systems. Moreover, the probe presented a high selectivity to *P. jirovecii*, since no response was observed in the presence of DNA from other microorganisms. Finally, the gated material **S2** is tested in unmodified sputum and bronchoalveolar lavages samples from infected and non-infected patients to diagnose PcP, obtaining a sensitivity and a selectivity of 100%. In the same way, **S2** is used to analyze nasopharyngeal aspirate samples from infants to detect primary infected individuals with a sensitivity and a selectivity of 70% and 78%, respectively. In both cases, **S2** detects *P. jirovecii* in 60 min without any sample treatment or the use of amplification reactions. This diagnostic tool has great potential for the further development of point-of-care devices for the simple and accurate detection of *P. jirovecii* from different biofluids avoiding PCR and using a small amount of sample, which could represent a high gain in the fight against PcP.

## Figures and Tables

**Table 1:** Sequences of the used oligonucleotides.

Oligonucleotide		Sequence (5′-3′)
**O1**	Duplex antiparallel	5′-GAAAGGGAAACAGCCCAG-3′
**O2**	Clamp antiparallel	5′-GACAAAGGGAAAG-TTTT-GAAAGGGAAACAGCCCAG-3′
**O3**	Control clamp antiparallel	5′-AGAGCAGAAAGGA-TTTT-GAAAGGGAAACAGCCCAG-3′
**O4**	Target complementary	5′-CTGGGCTGTTTCCCTTTC-3′

**Table 2:** Atomic elements ratio in the different prepared solids by energy dispersive X-ray spectroscopy (EDX) studies.

	C/Al	N/Al	P/Al
**S0**	1.52	0.38	-
**S1**	0.76	0.40	0.01
**S2**	0.58	0.53	0.02
**S3**	0.74	0.42	0.02

**Table 3:** Detection of *P. jirovecii* in clinical samples using the reference method (PCR) and **S2** material. Each sample was analyzed by triplicate.

Sample ^a^	Biological Fluid	Reference Method (PCR) ^b^		S2 ^c^
		Ct	Result	Result
1	Sputum	28	Infected	+
2	Sputum	27.5	Infected	+
3	BAL	36.6	Infected	+
4	Sputum	28.5	Infected	+
5	BAL	38.8	Infected	+
6	BAL	19	Infected	+
7	Sputum	26.8	Infected	+
8	Sputum	30.28	Infected	+
9	Sputum	35.3	Infected	+
10	Sputum	34.9	Infected	+
11	BAL	> 40	Non-Infected	-
12	Sputum	> 40	Non-Infected	-
13	NPA	-	Non- Infected	-
14	NPA	-	Non- Infected	-
15	NPA	-	Non- Infected	-
16	NPA	33.6	Infected	+
17	NPA	37.2	Infected	+
18	NPA	34.9	Infected	+
19	NPA	28.8	Infected	+
20	NPA	-	Non- Infected	-
21	NPA	33.4	Infected	+
22	NPA	-	Non- Infected	-
23	NPA	-	Non- Infected	-
24	NPA	-	Non- Infected	-
25	NPA	-	Non- Infected	+
26	NPA	-	Non- Infected	+
27	NPA	-	Non- Infected	-
28	NPA	34.9	Infected	-
29	NPA	39.4	Infected	-
30	NPA	34.4	Infected	+
31	NPA	34.6	Infected	+
32	NPA	33.4	Infected	+
33	NPA	35.5	Infected	-

^a^ Samples 1 to 12 were from patients of Hospital Universitari i Politècnic La Fe and samples 13 to 33 were from newborn infant patients of Hospital Universitario Virgen del Rocío. ^b^ Samples were considered positives (+) when two different aliquots of each patient were positive by specific PCR for *P. jirovecii.* ^c^ Samples were considered positives (+) when the fluorescence signal at 585 nm (λ_exc_ = 555 nm) was higher than the average fluorescence of the negative controls plus three times the standard deviation.

## Supplementary Materials

The following are available online at https://www.mdpi.com/2309-608X/6/4/292/s1, Scheme S1: Scheme of the duplexes formed by oligonucleotides O1 and O3 with the target, and the triplex formed by oligonucleotide O2 and the target sequence, Table S1: Mass spectrometry analysis of the oligonucleotides, Figure S1: Amount of rhodamine B released from the pores of solids **S1** (A) and **S3** (B) when 1 nM of DNA from *P. jirovecii* was (a) absent and (b) present in a solution of hybridization buffer (20 mM Tris-HCl, 37.5 mM MgCl_2_, pH 7.5), Figure S2: Delivery of rhodamine B from material **S2** in buffer, sputum, BAL, and NPA fluids in the presence of complementary DNA from *P. jirovecii* (1 nM).
