# Supplementary material for: Triplex Hybridization-Based Nanosystem for the Rapid Screening of Pneumocystis Pneumonia in Clinical Samples
Source: J Fungi (Basel). 2020 Nov 17;6(4):292. doi: 10.3390/jof6040292 (PMC7712664; doi:10.3390/jof6040292)
Supplement: Supplementary file 1 [file jof-06-00292-s001.pdf]

Electronic Supplementary Information

Triplex Hybridization-based Nanosystem for the Rapid Screening of Pneumocystis Pneumonia in Clinical Samples

Luis Pla[1,2,3], Anna Aviñó[3,4], Ramón Eritja[4,3], Alba Ruiz-Gaitán[5], Javier Pemán[5], Vicente Friaza[6], Enrique J. Calderón[6,7], Elena Aznar*[3,1,2,8], Ramón Martínez-Máñez*[1,2,3,8] and Sara Santiago-Felipe[3,1,2]

[1] Instituto Interuniversitario de Investigación de Reconocimiento Molecular y Desarrollo Tecnológico, Universitat Politècnica de València, Universitat de València, Camino de Vera s/n, 46022, Valencia, Spain.
[2] CIBER de Bioingeniería, Biomateriales y Nanomedicina (CIBER-BBN).
[3] Unidad Mixta de Investigación en Nanomedicina y Sensores. Universitat Politècnica de València, Instituto de Investigación Sanitaria La Fe, Valencia, Spain.
[4] Institute for Advanced Chemistry of Catalonia (IQAC), CSIC, Jordi Girona 18-26, 08034, Barcelona, Spain.
[5] Grupo Acreditado de Infección Grave. Instituto de Investigación Sanitaria La Fe and Hospital Universitari i Politècnic La Fe, Avenida Fernando Abril Martorell, 46026, Valencia, Spain.
[6] Instituto de Biomedicina de Sevilla, Hospital Universitario Virgen del Rocío/Consejo Superior de Investigaciones Científicas/Universidad de Sevilla.
[7] Centro de Investigación Biomédica en Red de Epidemiología y Salud Pública (CIBERESP).
[8] Unidad Mixta UPV-CIPF de Investigación en Mecanismos de Enfermedades y Nanomedicina. Universitat Politècnica de València, Centro de Investigación Príncipe Felipe, Valencia, Spain.

Design of oligonucleotides and in silico analysis of the mitochondrial large-subunit of Pneumocystis jirovecii (mt LSU rRNA).

In this study it was used the gene encoding the mitochondrial large-subunit of *Pneumocystis jirovecii* (mtLSUrRNA). This sequence was analyzed in order to find sequences that are able to form triplex structures. We used Analysis by Triplex-Forming Oligonucleotide Target Sequence Search Tool. To form a stable triplex we need consecutive pyrimidines in the sequence to be analyzed. We found two interesting purine sequences that the corresponding complementary sequences were pyrimidines .The first one in blue contain 17 nucleotides and contain 3 mismatches or interruptions in the formation of the triplex, the second is shorter 13 nucleotides and only one interruption. We selected Target 2 (green) 5′-CTGTTTCCCTTTC-3′ because although it has 13 nucleotides it contains only 1 interruption in the polypyrimidine track (one G).

GTGTACGTTGCAAAGTACTCAGAAGAATTGTGGTAAGTAGTGAAATACAAATCGGAC
TAGGATATAGCTGGTTTTCTGCGAAAATTGTTTTGGCAAATTGTTTATTCCTCT**NAAA**
AATAGTAGGTATAGCACTGAATATCTCGAGGGAGTATGAAAATATTTATCTCA
GATATTTAATCTCAAAATAACTATTTCTTAAAATAAATAATCAGACTATGTGCG
ATAAGGTAGATAGTCGAAAGGGAAACAGCCCAGAACAGTAATTAAAGCTCCCY
AATTAATATTAAGTGAAATAAAAGTTGTTGGATATCTAAAACAGTTAAGAAGTGGGCTT
GGAAACAGCCAC

Duplex-forming oligonucleotide **O1** was designed to be complementary to the green 13 nucleotides polypyrimidine track and to 5 nucleotides more (in total 18 nucleotides). The addition of these 5 nucleotides was done to increase the affinity and specificity. BLAST analysis showed that the sequence was not found in the human genome. Triplex-forming clamp **O2** contained the same complementary sequence and the reverse Hoogsteen sequence after a tetrathymidine loop as described [35-40]. The control clamp **O3** contained the complementary sequence followed by a scrambled sequence unable to form triplex.

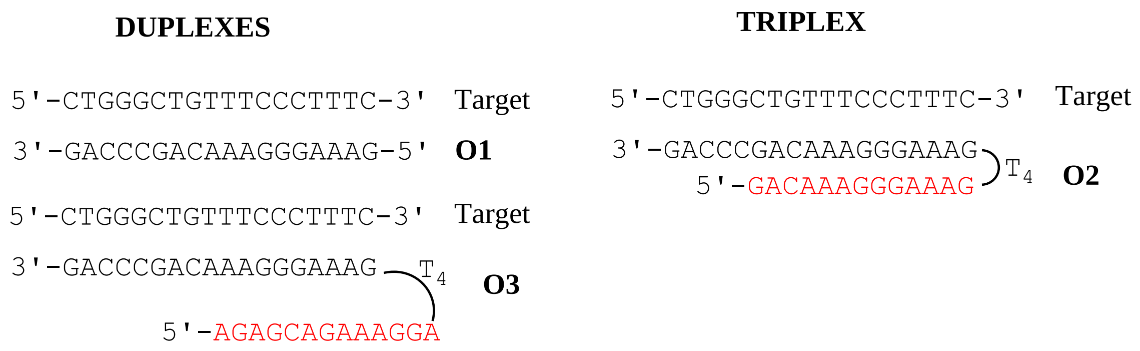

Scheme S1. Scheme of the duplexes formed by oligonucleotides **O1** and **O3** with the target, and the triplex formed by oligonucleotide **O2** and the target sequence.

Table S1. Mass spectrometry analysis of the oligonucleotides

Code	Name	M calculated (g/mol)	M found (g/mol)
**O1**	Duplex antiparallel	5575	5574
**O2**	Clamp antiparallel	10918	10915
**O3**	Control clamp antiparallel	10918	10912
**O4**	Target complementary	5423	5424

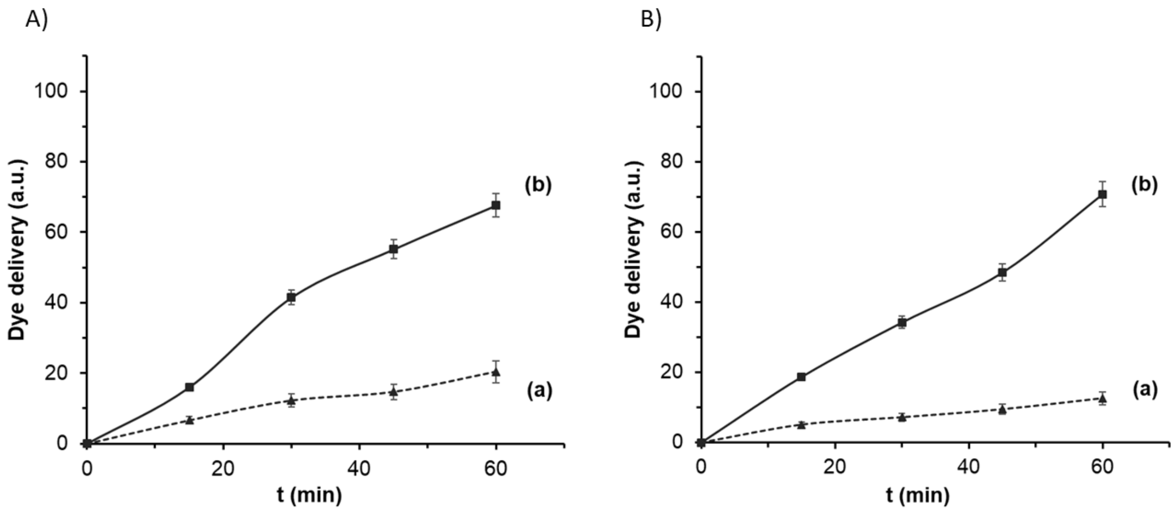

Figure S1. Amount of rhodamine B released from the pores of solids **S1** (A) and **S3** (B) when 1 nM of DNA from *P. jirovecii* was (a) absent and (b) present in a solution of hybridization buffer (20 mM Tris-HCl, 37.5 mM $MgCl_2$, pH 7.5).

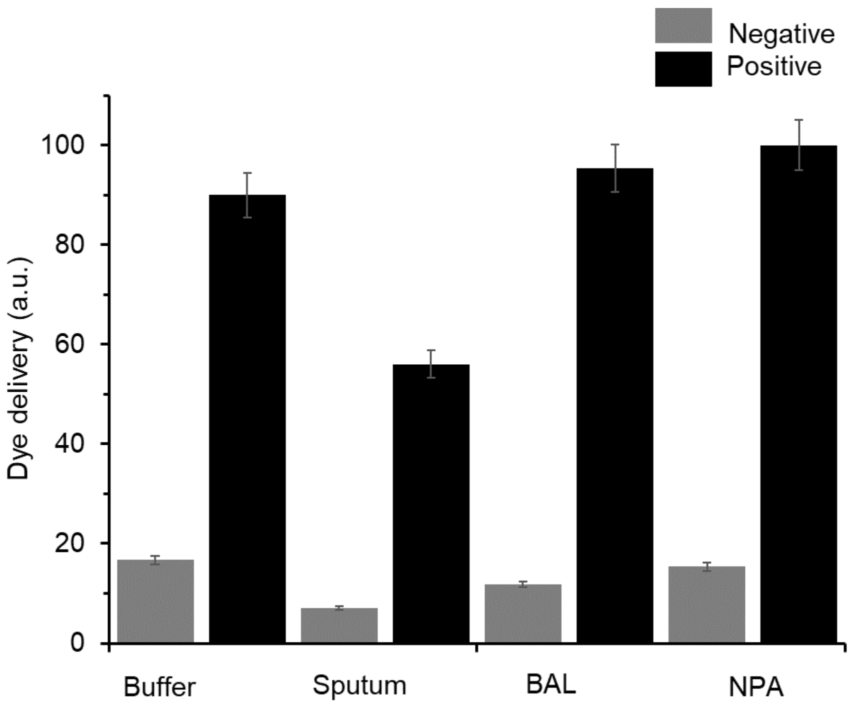

Figure S2. Delivery of rhodamine B from material **S2** in buffer, sputum, BAL and NPA fluids in the presence of complementary DNA from *P. jirovecii* (1 nM).
